# Interplay between hypoxia, RNA methylation, and HPV in head and neck squamous cell carcinomas: drivers of oncogenesis and resistance to therapy

**DOI:** 10.17179/excli2025-8834

**Published:** 2025-12-10

**Authors:** Marcel Mohr, Julia Kozikowska, Zuzanna Petryszyn, Kamila Ostrowska, Ewelina Golusinska-Kardach, Wojciech Golusinski, Wiktoria Suchorska, Katarzyna Kulcenty

**Affiliations:** 1 Doctoral School of Molecular Medicine, Medical University of Lodz, Haller Square 1, 90-624 Lodz, Poland; 2 Radiobiology Laboratory, Greater Poland Cancer Centre, Garbary 15 Street, 61-866 Poznan, Poland; 3 Department of Head and Neck Surgery, Poznan University of Medical Sciences, Garbary 15 Street, 61-866 Poznan, Poland; 4 Master Student at Adam Mickiewicz University, Uniwersytetu Poznańskiego 6 Street, 61-614 Poznan Poznan, Poland; 5 Department of Molecular Biology and Genetics, Aarhus University, Nordre Ringgade 1, 8000 Aarhus Centrum, Denmark; 6 Department of Dental Surgery, Periodontology and Oral Mucosa Diseases, Poznan University of Medical Sciences, Bukowska 70 Street, 60-812 Poznan, Poland; 7 Department of Electroradiology, Poznan University of Medical Sciences, Fredry 10 Street, 61-701 Poznan, Poland

**Keywords:** head and neck squamous cell carcinoma, human papilloma virus, N6-Methyladenosine (m6A) RNA modification, hypoxia, immunotherapy

## Abstract

Head and neck squamous cell carcinoma (HNSCC) encompasses a diverse group of tumors with varying etiology, biology, and response to therapy. Among its subtypes, human papillomavirus positive HNSCC is associated with better prognosis and enhanced sensitivity to radiotherapy, chemotherapy, and immunotherapy. However, resistance still occurs and is often driven by complex molecular mechanisms that remain incompletely understood. Recent evidence highlights the pivotal role of RNA modifications-particularly N6-methyladenosine (m⁶A)-in regulating key processes such as gene expression, immune response, and treatment resistance. Dysregulation of m⁶A machinery, including methyltransferases (METTL3, METTL14), demethylases (FTO, ALKBH5), and m⁶A readers (YTHDFs, IGF2BPs), has been implicated in oncogenesis, immune evasion, and therapy failure in multiple cancers, including HNSCC. These epitranscriptomic changes intersect with hypoxia-driven signaling pathways, which reshape the tumor microenvironment, promote immunosuppression, and impair DNA repair, further contributing to resistance to conventional and targeted therapies. Moreover, in HPV-related HNSCC, viral oncoproteins modulate both RNA methylation and host immune dynamics, creating a unique biological context where m⁶A modifications may serve as mediators of HPV-specific oncogenic programs and therapeutic vulnerabilities. This review integrates current knowledge on the interplay between hypoxia, m⁶A RNA methylation, and HPV infection in HNSCC, emphasizing their combined role in shaping tumor progression and resistance. A deeper understanding of these pathways may offer new opportunities for biomarker discovery and the development of rational combination therapies.

See also the graphical abstract(Fig. 1).

## Abbreviations

BER: Base Excision Repair

CSC: Cancer Stem Cell

CTLA-4: Cytotoxic T-Lymphocyte-Associated Protein 4

DC: Dendritic Cell

DC1: YTHDC1 

DC2: YTHDC2

DF: YTHDF Protein Family

DF1: YTHDF1

DF2: YTHDF2

DF3: YTHDF3

DSB: Double-Strand Break

ECM: Extracellular Matrix

EGFR: Epidermal Growth Factor Receptor

EMT: Epithelial-Mesenchymal Transition

FA: Fanconi Anemia DNA Repair Pathway

FDA: Food and Drug Administration

FTO: Fat Mass And Obesity-Associated Protein

HIF: Hypoxia-Inducible Factor

HIF-1α: Hypoxia-Inducible Factor 1 α Subunit

HIF-1β: Hypoxia-Inducible Factor 1 β Subunit

HNSCC: Head And Neck Squamous Cell Carcinoma

HPV: Human Papillomavirus

HR: Homologous Recombination

HRE: Hypoxia Response Element

ICL: Interstrand Crosslinks

IL-10: Interleukin-10

LSCC: Laryngeal Squamous Cell Carcinoma

M1: M1-Polarised Macrophages

M2: M2-Polarized Macrophages

m³U: 3-Methyluridine

m^6^A: N6-Methyladenosine

MDSC: Myeloid-Derived Suppressor Cell

MHC I: Major Histocompatibility Complex Class I

MHC II: Major Histocompatibility Complex Class II

MHC: Major Histocompatibility Complex

MRP: Multidrug Resistance Protein

ncRNA: non-coding RNA

NER: Nucleotide Excision Repair

NHEJ: Non-Homologous End Joining

NK: Natural Killer Cell

NPC: Nasopharyngeal Carcinoma

OPSCC: Oropharyngeal Squamous Cell Carcinoma

ORR: Overall Response Rates

OSCC: Oral Squamous Cell Carcinoma

P-bodies: RNA processing bodies

PD-1: Programmed Death Protein 1

PDH: Prolyl Hydroxylase

PD-L1: Programmed Death-Ligand 1

pVHL: Von Hippel-Lindau Protein

R/M: Reccurent/Metastatic

Rb: Retinoblastoma Protein

ROS: Reactive Oxygen Species

shRNA: Short hairpin RNA

snRNA: Small Nuclear RNA

SNSCC: Sinonasal Squamous Cell Carcinoma

SRSF: Serine/Arginine-Rich Splicing Factor

SSB: Single-Strand Break

TCA: Tricarboxylic Acid

TGF-β: Transforming Growth Factor Beta

TIL: Tumor Infiltrating Lymphocyte

TLR: Toll-Like Receptor

TME: Tumor Microenvironment

Treg: Regulatory T Cell

## Epidemiology and Risk Factors of HNSCC

Head and neck squamous cell carcinoma, the sixth most common cancer worldwide, develops from mucosal epithelium of tongue, oral cavity, pharynx or larynx. The principal risk factors of HNSCC are tobacco use, alcohol consumption and human papillomavirus (HPV) infection. HNSCC of oral cavity and larynx are linked to tobacco and alcohol exposure, whereas oropharyngeal HNSCC is predominantly correlated with HPV infection (Jiang et al., 2024[41]). Among the oncogenic HPV types, HPV-16 and HPV-18 are chiefly implicated in HNSCC, with HPV-16 responsible for almost 60 % of oropharyngeal cancers (Galati et al., 2024[26], Johnson et al., 2020[42]).

The survival rate for HNSCC is approximately 40-50 % despite the development of various treatments, including surgery, chemo-/radio-/immunotherapy. The poor prognosis of patients is caused by late diagnosis, as T4 is the most common stage at diagnosis for oral and oropharyngeal cancer (Gormley et al., 2022[30]).

## HPV and HNSCC: A Changing Epidemiological Profile

Over the years there has been an increase of HPV-positive HNSCC, particularly among young people and those not using tobacco and alcohol (Deschler et al., 2014[13]). HPV-negative HNSCC is much more common in the older population not abstaining from such substances (Farsi et al., 2017[21]). The increased incidence of HPV-positive HNSCC among younger, non-smoking and non-drinking individuals indicates another important risk factor for HNSCC that is sexual transmission of HPV. Oral sex is a key mode of HPV transmission and the changing sexual behaviour of recent decades has contributed to HNSCC (Shah et al., 2017[81]).

Epidemiological analyses of the U.S. population from 1992 to 2015 have demonstrated a significant increase in the incidence of oropharyngeal squamous cell carcinoma (OPSCC), particularly among white males, with an annual increase of 3.8 %. This rising trend has been characterized as a virus-associated epidemic primarily affecting younger individuals, with projections estimating approximately 30,000 new cases per year by 2029. Notably, the highest incidence rate has been observed among individuals born before 1955, with a 5.3 % annual increase (Tota et al., 2019[91]).

This demographic trend is largely attributed to historically lower awareness of safe sexual practices in these cohorts, primarily due to the lack of comprehensive sex education in the pre-HIV era, which facilitated the transmission of HPV. As the population continues to age, the burden of OPSCC is expected to shift towards older white males. This shift underscores the need for treatment strategies that are better tailored to the health status and comorbidities of an ageing patient population (Tota et al., 2019[91]).

Over the next two decades, a significant increase in HPV-positive HNSCC is anticipated, particularly in developed regions such as Europe, North America, Australia, New Zealand (de Martel et al., 2017[12]). The change in demographic profile points to the increased importance of HPV vaccination and public awareness programmes to reduce the increasing number of HPV-dependent cancers. Current HPV-16 and HPV-18 vaccines as a prevention for cervical cancer are able to potentially reduce the incidence of HPV-positive HNSCC; however, differences in the risk factor profiles and pathogenesis mechanisms of the two diseases indicate the need for novel diagnostic and therapeutic strategies (Olsson et al., 2007[66]).

HPV-positive HNSCC patients have a better response to treatment (radio-,chemotherapy), and higher overall survival rates (Li et al., 2018[48]). Data showed a significant increase in three-year overall survival rates of 82.4 % in HPV-positive compared to 57.1 % in HPV-negative HNSCC patients (Ang et al., 2010[1]). HPV status has become a key factor in the treatment stratification of HNSCC. The better prognosis for patients affected by HPV-positive HNSCC has led to the development of new treatment regimens, improving quality of life by reducing treatment-related toxicity (Wang et al., 2019[95]). The inclusion of HPV status in the disease staging system and treatment guidelines has resulted in increased accuracy of prognosis and clinical decision-making (Lewis et al., 2018[46]). Better treatment response and prognosis of HPV-positive HNSCC have been attributed to several factors. Firstly, stronger protection from the immune system may be caused by patients' healthier lifestyles, as they are less likely to be heavy smokers or alcohol abusers (Deschler et al., 2014[13]). Furthermore, the presence of HPV may trigger an enhanced immune response resulting in a better prognosis (Chakravarthy et al., 2016[7], Wang et al., 2019[95]). Thirdly, the characteristics of HPV-positive tumors, i.e. less aggressiveness and greater sensitivity to radiotherapy, translate into better patient outcomes (Mirghani et al., 2015[59]). 

## HPV-Driven Cellular Transformation

**Figure 2:**
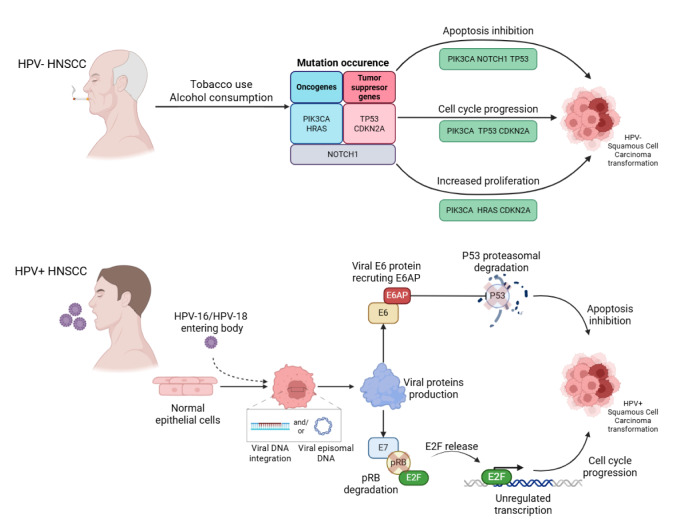
Molecular pathways in HPV-negative and HPV-positive HNSCC. In HPV-negative HNSCC (top panel), chronic exposure to carcinogens such as tobacco and alcohol leads to the accumulation of somatic mutations in key oncogenes (*PIK3CA*, *HRAS*) and tumor suppressor genes (*TP53*, *CDKN2A*, *NOTCH1*). These genetic alterations disrupt normal cellular regulation by inhibiting apoptosis, promoting cell cycle progression, and enhancing proliferation, ultimately resulting in malignant transformation of epithelial cells. In contrast, HPV-positive HNSCC (bottom panel) is driven by infection with high-risk HPV (e.g., HPV-16/18). Following viral entry into epithelial cells, the HPV genome integrates into the host DNA or persists as episomal DNA, leading to the expression of viral oncoproteins E6 and E7. E6 promotes proteasomal degradation of the tumor suppressor p53. E7 protein inactivates pRB, releasing E2F transcription factors and triggering unregulated transcription and uncontrolled cell cycle progression, driving tumorigenesis.

Human papillomaviruses are highly epitheliotropic DNA viruses, which targets the multilayered keratocyte regions in order to replicate (Speel, 2017[87]). The viral genome encodes two genes (*L1* and *L2*) necessary for infection and six genes (*E1*-*E7*) involved in viral replication, regulation, and HPV-related cellular transformation (Pinkiewicz et al., 2022[73]). The oncogenic potential of HPV-driven cancers primarily stems from the viral proteins E6 and E7, which disrupt crucial tumor suppressor pathways (Williams et al., 2011[105]). E6 facilitates the degradation of p53, a key regulator of DNA damage response and apoptosis, by recruiting the cellular ubiquitin ligase E6AP (Mijit et al., 2020[57]). The loss of p53 function allows cells with genetic abnormalities to continue dividing rather than undergoing programmed cell death. E7 protein, on the other hand, targets the retinoblastoma (Rb) protein for degradation, leading to unchecked activation of E2F transcription factors, which drive the cell cycle forward regardless of regulatory signals (McLaughlin-Drubin and Münger, 2009[56]). Together, these alterations result in uncontrolled cellular proliferation, increased genomic instability, and a weakened ability to undergo cell cycle arrest in response to damage (Figure 2(Fig. 2)). 

The expression of cyclin D1, protein encoded by *CCND1* gene, involved in cell cycle regulation is a key distinction between HPV-positive and HPV-negative tumors. In HPV-negative cancers, Rb function is not disrupted by viral oncoproteins leading to *CCND1* overexpression and excessive cell cycle progression and enhanced tumor growth. By contrast, HPV-positive tumors are characterized by *CCND1* inhibition through E7-mediated Rb inactivation (Farah, 2021[20]). Additionally, HPV-negative tumors frequently exhibit mutations in the *CDKN2A* gene, which encodes, a protein that normally inhibits cyclin-dependent kinases and restricts cell cycle progression. In HPV-positive cancers, p16 is paradoxically overexpressed as a compensatory response to E7-mediated Rb degradation, constituting p16 as a useful biomarker for distinguishing HPV-related tumors (Farah, 2021[20]).

Beyond cell cycle deregulation, HPV-positive tumors also develop sophisticated mechanisms to evade immune detection. By suppressing the expression of major histocompatibility complex (MHC) molecules, HPV-positive tumors reduce their visibility to the host immune system (Westrich et al., 2017[103]). Additionally, increased expression of the immune checkpoint programmed death-ligand 1 (PD-L1) protein inhibits T-cell activation, allowing cancer cells to persist and evade immune surveillance (Lim et al., 2019[51]). These features explain why HPV-positive tumors tend to respond better to immune checkpoint inhibitors, such as anti- programmed death protein 1 (PD-1) and anti-PD-L1 therapies, which aim to restore the patients' immune system's ability to recognize and destroy cancer cells.

The differences in genomic landscape of HPV-positive and HPV-negative HNSCC emphasize the diverse nature of those tumors. HPV-negative HNSCC genetic alterations frequently involve direct mutations in the *TP53* gene. More than 70 % of HPV-negative HNSCCs harbor *TP53* mutations, leading to the loss of p53 function and contributing to aggressive tumor behavior and poorer treatment responses. In contrast, HPV-positive HNSCC rarely exhibit *TP53* mutations since p53 is already functionally inactivated by viral protein E6 (Mountzios et al., 2014[61]). Instead, they frequently harbor mutations in the *PIK3CA* gene, which encodes a critical regulator of the PI3K/AKT/mTOR signaling pathway. This pathway plays a central role in cell survival, metabolism, and tumor progression, making *PIK3CA* a promising target for therapeutic intervention in HPV-driven malignancies (Lim et al., 2019[51], Mountzios et al., 2014[61]). 

The presence of these distinct genetic alterations significantly influences treatment responses and therapeutic strategies. HPV-positive tumors, characterized by lower mutational burdens and immune evasion mechanisms, tend to be more sensitive to radiation therapy and immune checkpoint blockade. In contrast, HPV-negative tumors, with frequent *TP53* mutations, *CCND1* amplification, and epidermal growth factor receptor (EGFR) overexpression, are often more resistant to standard therapies and may require combination treatments involving chemotherapy, EGFR inhibitors, or targeted molecular agents (Chung et al., 2015[11], Farah, 2021[20], Mountzios et al., 2014[61], Shi et al., 2023[82]). The growing understanding of these molecular distinctions continues to shape personalized treatment approaches, highlighting the importance of tailoring therapies based on the underlying genetic and viral characteristics of each tumor.

## The Role of Hypoxia in HNSCC Progression and Therapy Resistance

Hypoxia is a characteristic feature observed in up to 90 % of solid tumors, including HNSCC, and is recognized as a hallmark of cancer (Chen et al., 2023[9]). The tumor microenvironment (TME) in solid malignancies is composed of heterogeneous populations, including cancer cells, immune cells, cancer stem cells (CSCs), fibroblasts, extracellular matrix (ECM) components, and blood and lymphatic vessels. This complex structure leads to uneven oxygen distribution, particularly in the tumor core, where high cellular density and metabolic demand exceed the oxygen supply provided by dysfunctional vasculature (Emami Nejad et al., 2021[19]).

Cellular adaptation to hypoxia is largely governed by hypoxia-inducible factors (HIFs), heterodimeric transcription factors consisting of an oxygen-regulated α-subunit (commonly HIF-1α) and a constitutively expressed β-subunit (HIF-1β) (Chen et al., 2023[9]). Under normoxic conditions, HIF-1α is hydroxylated by prolyl hydroxylases (PHD1-3) and targeted for ubiquitin-mediated degradation via the von Hippel-Lindau (pVHL) protein (Ostapowicz et al., 2024[67]). Under hypoxic or iron-depleted conditions, HIF-1α is stabilized, translocates to the nucleus, and dimerizes with HIF-1β. The active complex binds to hypoxia response elements (HREs) in target gene promoters, activating pathways that promote cellular adaptation to low oxygen (Ostapowicz et al., 2024[67]).

HIF-1α directly upregulates genes involved in angiogenesis, such as *VEGF*, *SDF1*, and *ANGPT2*, which promote the formation of abnormal, leaky blood vessels that further impair oxygen and drug delivery (Rey and Semenza, 2010[75]). Interestingly, *VEGF* expression is significantly lower in HPV-positive HNSCC than in HPV-negative tumors, suggesting that HPV status may influence the angiogenic profile of HNSCC (Baruah et al., 2015[4]).

Hypoxia also reprograms cellular metabolism by promoting anaerobic glycolysis, known as the Warburg effect (Icard et al., 2018[38]). HIF-1α upregulates *GLUT1*, enhancing glucose uptake, and several glycolytic enzymes including PDK1, LDHA, and PKM. PDK1 inhibits PDH, blocking pyruvate conversion to acetyl-CoA and entry into the tricarboxylic acid (TCA) cycle, thereby promoting glycolysis. LDHA converts pyruvate to lactate, contributing to extracellular acidosis, while PKM2 facilitates metabolic adaptation favoring proliferation and survival (Wicks and Semenza, 2022[104]).

CSCs phenotype is also considered to be maintained by hypoxia conditions. HNSCC stem-like cells are characterized by upregulation of stemness markers including OCT3, OCT4, SOX2 and NANOG (Johnson et al., 2020[42]), which expression promotes invasiveness in HNSCC cells (Emami Nejad et al., 2021[19]). Furthermore, stem-like phenotype is strongly linked to epithelial-mesenchymal transition (EMT) characterized by loss of cell-cell interactions, gaining mesenchymal cell features, detachment from primary tumor or remodeling ECM (Emami Nejad et al., 2021[19]). HIF1 induces expression of SNAIL and TWIST transcription factors that promote EMT through E-cadherin downregulation (Johnson et al., 2020[42]). Therefore, loss of E-cadherin has been linked to increased metastasis in HNSCC (Peltanova et al., 2019[72]). Altogether, weak extracellular interactions and additional vessels are a great opportunity for cancer cells migration and metastasis, highlighting the role of hypoxia signaling in tumor progression.

Therapeutically, hypoxia reduces the effectiveness of radiotherapy by limiting oxygen availability for the formation of DNA-damaging reactive oxygen species (ROS) and promoting DNA repair mechanisms (Chen et al., 2023[9]). It also contributes to chemoresistance through several mechanisms: HIF-1α induces *MDR1* expression, encoding P-glycoprotein that actively effluxes drugs from cancer cells; lactate accumulation lowers extracellular pH, impairing the activity of pH-sensitive drugs (e.g., anthracyclines); and poorly perfused tumor vessels prevent efficient drug delivery (Kopecka et al., 2021[45]). Moreover, the lack of oxygen directly diminishes the cytotoxic efficacy of drugs like cisplatin and 5-fluorouracil (Rohwer et al., 2010[77]).

Hypoxia also impairs anti-tumor immune responses. HIF-1α upregulates immune checkpoints such as PD-L1, PD-1, and cytotoxic T-lymphocyte-associated protein 4 (CTLA-4) on tumor and immune cells, respectively, leading to immune evasion (Hill et al., 2022[32]). Furthermore, the hypoxic, acidic TME favors recruitment of immunosuppressive cells including M2-polarized macrophages (M2), myeloid-derived suppressor cells (MDSCs), and regulatory T cells (Tregs), while limiting the infiltration and activity of CD8⁺ T cells, which are essential for effective anti-tumor immunity (Kopecka et al., 2021[45]).

Clinically, increased HIF-1α expression correlates with poor prognosis, higher mortality, and resistance to radiotherapy in HNSCC patients (Semenza, 2007[80]). Paradoxically, studies have shown that HIF-1α protein levels are higher in HPV-positive oropharyngeal HNSCC cell lines compared to HPV-negative lines derived from the oral cavity, under both normoxic and hypoxic conditions (Knuth et al., 2017[44]). This contradicts the generally favorable outcomes associated with HPV-positive HNSCC. Additional studies, such as those involving sinonasal squamous cell carcinoma (SNSCC), have failed to show consistent correlations between HPV status and HIF-1α, VEGF, or GLUT1 expression, suggesting that the impact of HPV on hypoxia signaling may be tumor subtype-specific (Vinciguerra et al., 2023[92]).

Overall, hypoxia-driven HIF-1α signaling is a central mediator of tumor progression, treatment resistance, and immune evasion in HNSCC. Its interplay with HPV infection and m⁶A RNA methylation warrants further investigation as a potential target for improving therapy outcomes in this disease.

## m6A Modifications: Writers, Readers, and Erasers - Mechanism of Action

**Figure 3:**
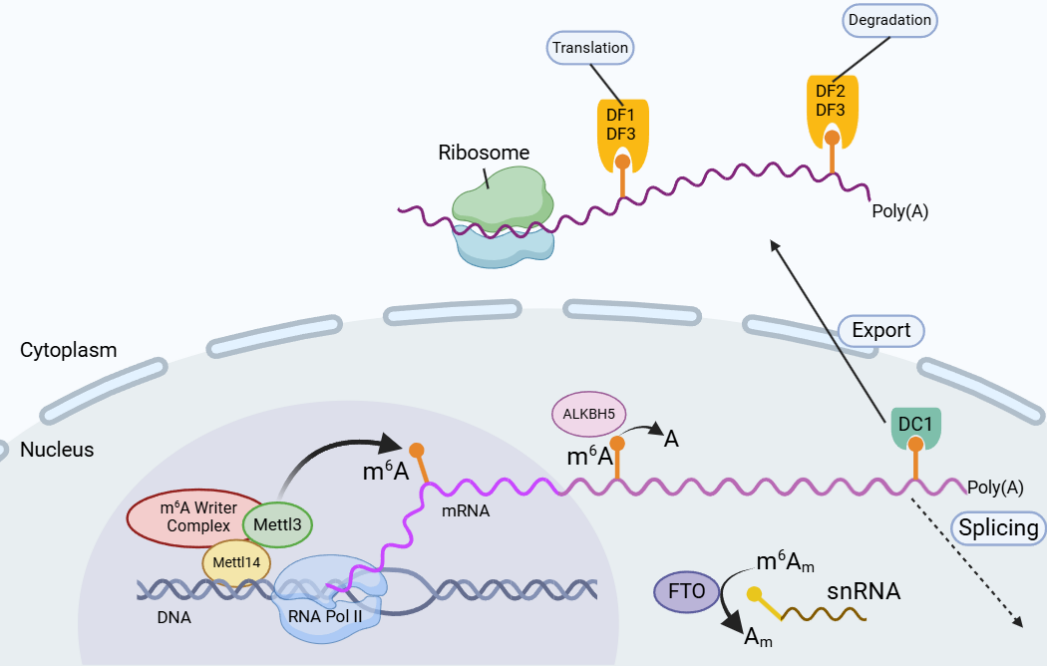
mRNA m^6^A epigenetic modifications. The cycle of an mRNA undergoing m^6^A modification begins in the nucleus during transcription. The m^6^A writer complex, which includes the core METTL3 and its associated protein METTL14, facilitates this modification co-transcriptionally. Additionally, m^6^A erasers, such as ALKBH5, are primarily localized in the nucleus, where they remove m^6^A signatures. Interestingly, the FTO has been found to selectively target m^6^Am rather than m^6^A, with its primary substrate located in small nuclear RNAs (snRNAs). Within the nucleus, m^6^a modifications can interact with specific nuclear reader proteins, particularly DC1, potentially influencing splicing and other nuclear processes like mRNA export. Once exported to the cytoplasm, m^6^a modifications bind to distinct reader proteins that regulate mRNA stability, translation, and localization. In the cytoplasm, m^6^A readers such as DF1 and DF3 promote translation of m^6^A-marked mRNAs. Meanwhile, DF2 and DF3 facilitate the degradation of m^6^A -modified transcripts.

The regulation of mRNA fate through dynamic m⁶A modification involves a coordinated interplay between methyltransferases, demethylases, and reader proteins operating across nuclear and cytoplasmic compartments (Figure 3(Fig. 3)).

### m^6^A writers

The deposition of m⁶A in mRNA is mediated by a multi-subunit methyltransferase complex with high specificity. Only a subset of mRNAs undergoes this modification, and even within these transcripts, only a fraction of potential m⁶A consensus motifs are methylated. The precise determinants of both transcript- and site-specificity remain incompletely understood. While much attention has been given to m⁶A in mRNA, the majority of m⁶A modifications in cellular RNA are actually found in ribosomal RNA, which is far more abundant. In mammals, four methyltransferases are responsible for depositing m⁶A across different RNA species. The primary writer complex for mRNA and other RNA polymerase II-derived transcripts is the METTL3-METTL14 heterodimer, where METTL3 provides catalytic activity, while METTL14 functions as an allosteric regulator and RNA-binding partner (Śledź and Jinek, 2016[86], Wang et al., 2016[96]). Among methyltransferases, METTL3-METTL14 plays the dominant role in m⁶A deposition within mRNA. Genetic knockout of *METTL3* or CRISPR-mediated disruption of *METTL14* in mouse embryonic stem cells results in the loss of more than 99 % of m⁶A from polyadenylated RNA, (Geula et al., 2015[28]) demonstrating that only a small fraction of m⁶A sites in poly(A) RNA are attributed to METTL16 or other potential methyltransferases. Because of this, *METTL3* and *METTL14* deletions have been widely employed to investigate the functional consequences of m⁶A modification (Batista et al., 2014[5], Geula et al., 2015[28]). 

### m^6^A readers

The effects of m⁶A on mRNA stability, localization, and translation are largely mediated by specialized m⁶A-binding proteins, known as readers. These proteins recognize and interact with m⁶A-marked transcripts, influencing their fate within the cell. The first identified m⁶A readers were proteins containing the YTH domain, which provided critical insight into how m⁶A exerts its regulatory functions. Additionally, m⁶A can destabilize local RNA secondary structures, indirectly altering the binding affinity of various RNA-binding proteins (Dominissini et al., 2012[16]). 

In mammals, five YTH domain-containing proteins are classified into three groups: YTHDC1 (DC1), YTHDC2 (DC2), and the YTHDF (DF) protein family. These proteins are distributed in different cellular compartments, reflecting their distinct functions. YTHDC1 is predominantly found in the nucleus, where it regulates mRNA processing and export (Hartmann et al., 1999[31]). The YTHDF proteins primarily function in the cytoplasm, where they influence mRNA degradation and translation (Li et al., 2017[47], Wang et al., 2015[99]). YTHDC2, in contrast, exhibits both nuclear and cytoplasmic localization, suggesting a broader regulatory role (Wojtas et al., 2017[106]). 

DC1 is a nuclear m⁶A-binding protein that plays a role in mRNA splicing (Xiao et al., 2016[109]), nuclear export (Roundtree et al., 2017[78]), and gene silencing (Patil et al., 2016[70]). It localizes to nuclear speckles, which are dynamic subnuclear structures enriched in RNA-processing factors. Initially observed in punctate "YT bodies", (Nayler et al., 2000[63]) these structures were later recognized as nuclear speckles due to their colocalization with serine/arginine-rich splicing factors (SRSFs) (Xiao et al., 2016[109]). DC1 also contains a low-complexity domain, similar to its cytoplasmic counterparts, suggesting that phase separation may be important for its function (Patil et al., 2016[70]). 

DC1 is thought to interact with newly transcribed mRNAs, binding them soon after m⁶A modification occurs. It partners with SRSF3, a splicing regulator that enhances exon inclusion, potentially by competing with another splicing factor, SRSF10 (Xiao et al., 2016[109]). 

The DF protein family consists of three closely related members: YTHDF1 (DF1), YTHDF2 (DF2), and YTHDF3 (DF3) (Patil et al., 2018[71]). These proteins share high sequence identity, particularly in their YTH domains, which directly bind m⁶A-modified mRNAs. The remainder of each protein consists of a low-complexity, prion-like region, which enables them to undergo phase separation upon mRNA binding (Patil et al., 2018[71]). This property allows them to recruit modified transcripts into cellular RNA-protein condensates, such as processing bodies (P-bodies) and stress granules, where mRNA fate is determined (Ries et al., 2019[76]). 

### m⁶A erasers

RNA modifications, once thought to be permanent, are now recognized as dynamic and reversible, thanks to enzymes capable of removing m⁶A from transcripts (Jia et al., 2011[39]). These demethylases, often referred to as m⁶A erasers, were initially believed to play a crucial role in turning gene expression by rapidly adjusting m⁶A levels in response to cellular signals (Zhao et al., 2017[116]). However, contrary to early assumptions, m⁶A turnover appears to be relatively stable under normal physiological conditions. 

The enzyme FTO (fat mass and obesity-associated protein) was the first demethylase identified, originally linked to the ALKB family of dioxygenases, which function in the repair of alkylated DNA and RNA bases (Fedeles et al., 2015[22], Gerken et al., 2007[27]). Initial studies suggested that FTO had limited activity toward methylated DNA nucleotides, but further biochemical assays revealed that it could demethylate 3-methyluridine (m³U) in RNA, sparking interest in its potential role in epitranscriptomic regulation (Gerken et al., 2007[27], Jia et al., 2008[40]). Later research confirmed that FTO could act on m⁶A, leading to its classification as an m⁶A eraser (Jia et al., 2011[39]). 

A breakthrough in understanding FTO's function came with the discovery that its true preferred substrate is not m⁶A, but m⁶Am, a related methyl modification found exclusively at the 5′ cap of mRNA (Mauer et al., 2017[55]). 

In contrast, ALKBH5, the second identified m⁶A eraser, has been firmly established as a genuine m⁶A demethylase (Zheng et al., 2013[118]). ALKBH5 exhibits strong substrate specificity for m⁶A-modified RNA. Localized predominantly in the nucleus, ALKBH5 appears to regulate m⁶A levels in nuclear transcripts, potentially influencing mRNA processing before it is exported to the cytoplasm (Zheng et al., 2013[118]). Its clear enzymatic activity, ALKBH5 does not appear to be essential for normal development, as knockout mice exhibit no major physiological abnormalities, except for severe defects in spermatogenesis (Zheng et al., 2013[118]). 

ALKBH5 has been implicated in cancer progression. In certain cancers, ALKBH5 expression is significantly upregulated, potentially altering m⁶A-dependent gene regulation to promote tumor growth (Dixit et al., 2017[14], Zhang et al., 2017[115]). One mechanism by which this occurs is through the hypoxia-inducible factor HIF-1α, which directly binds the ALKBH5 promoter, driving its expression in response to low oxygen conditions (Thalhammer et al., 2011[90]). Additionally, ALKBH5 levels increase following viral infections, raising the possibility that it plays a broader role in regulating RNA modifications during cellular stress responses (Rubio et al., 2018[79]). 

Currently, it is known that multiple proteins involved in m^6^A RNA modification may regulate oncogenes and suppressor genes in various cancers (Chen et al., 2022[10]). Deregulation of enzymes involved in m^6^A RNA modification is observed in HNSCC. In oral squamous cell carcinoma *METTL3*, *METTL14*, *FTO*, *YTHDF1*, *YTHDF2*, *YTHDC1* and *YTHDC2* relative mRNA expression is upregulated compared to normal tissue, but *ALKBH5* expression was unchanged. What is more, a higher *METTL3* expression was positively associated with advanced tumor stage, increased tumor cell proliferation and enhanced migration (Liu et al., 2020[52]). 

## Interplay Between m6A mRNA Methylation and HPV in HNSCC Progression and Treatment Response

RNA methylation is a critical mechanism involved in the regulation of genes and cellular processes, significantly influencing cancer biology (Sun et al., 2019[89]). The mechanisms regulating RNA methylation in the presence of HPV are mainly driven by the viral oncoproteins E6 and E7 (Zheng et al., 2022[120]). These proteins are able to interact with the cellular machinery to modify the epigenetic landscape. The HPV E6 protein affects the activity of methyltransferases (Hsu et al., 2012[34]). As a result of methyltransferases activity, the stability, splicing and translation of various mRNA molecules may be altered, resulting in induction of oncogenic processes in HPV-positive HNSCC (Maity and Das, 2016[54]). The E7 protein, as a result of targeting RB and interfering with cell cycle regulation, indirectly causes an effect on RNA methylation by altering the expression of regulatory genes responsible for RNA processing and modification (Fu et al., 2014[25], Slebos et al., 1994[85]). 

Overexpression of METTL3 or reduced activity of ALKBH5 has been linked to enhanced mRNA methylation, resulting in the stabilization of transcripts involved in cell proliferation and survival. Conversely, loss of m^6^A modifications may impair the proper degradation of oncogenic mRNAs or disrupt cellular differentiation (Wang et al., 2014[98]). In oral squamous cell carcinoma (OSCC), both *METTL3* and *METTL14* are significantly overexpressed in cancerous tissues compared to normal oral tissues, with higher expression levels correlated with advanced tumor stages and poorer histopathological differentiation. This dysregulation results in an altered mRNA methylation status, affecting the stability and translation of key oncogenes and tumor suppressor genes. *In vitro *experiments show that silencing *METTL3* and *METTL14* in OSCC cell lines inhibits cell proliferation, further supporting their role in tumor growth. Additionally, overexpression of these enzymes is associated with shorter overall survival in OSCC patients, positioning METTL3 and METTL14 as independent prognostic markers (Wang et al., 2016[97], Wu et al., 2022[108]). 

Distinct patterns of m^6^A methylation are present in HPV-positive tumors, which correlate with specific gene expression profiles and clinical outcomes. Altered m^6^A methylation in HPV-positive HNSCC leads to increased proliferation and resistance to apoptosis of tumour cells (Ban et al., 2020[3]). Writer METTL14 and eraser ALKBH5 collaboratively modulate m^6^A levels on transcripts that control key processes including cell cycle progression, EMT, and angiogenesis. Additionally, hypoxia disrupts the balance of m^6^A machinery by altering the expression of *METTL14*, *ALKBH5*, and the m^6^A reader *YTHDF3*, leading to reduced m^6^A methylation and aberrant gene expression. This disruption contribute to uncontrolled tumor growth, invasion, and angiogenesis (Panneerdoss et al., 2018[69]). 

In the case of HPV-positive HNSCC, an immune-invasive phenotype is noticeable, resulting in part from modifications of RNA methylation in immune-related genes. This regulation may translate into altered cytokine production, immune cell infiltration and expression of immune checkpoints, affecting the overall immune landscape of the tumor (Feng et al., 2021[23], Yi et al., 2020[111]). 

### Radiotherapy

HPV-positive HNSCC is considered more responsive to radiotherapy due to its inherent DNA repair deficiencies, certain tumors develop mechanisms of adaptive resistance that allow them to evade radiation-induced cell death (Kimple et al., 2013[43]). HPV-positive tumors exhibit unique DNA repair deficiencies due to the expression of E6 and E7 oncoproteins. One of the most significant consequences of HPV-driven genomic instability is the defective repair of radiation-induced double-strand breaks (DSBs). Homologous recombination (HR), a high-fidelity DNA repair pathway that operates in the S and G2 phases of the cell cycle, is compromised in HPV-positive tumors due to defective recruitment of BRCA2 and RAD51 to DNA damage sites. This defects initially contributes to the radiosensitivity observed in many HPV-positive HNSCC, as cells struggle to repair DSBs efficiently. The alternative DNA repair pathway is non-homologous end joining (NHEJ), which can lead to error-prone repair and the accumulation of mutations that promote tumor survival post-radiotherapy (Weaver et al., 2015[101]). Compensatory mechanisms, such as upregulation of base excision repair (BER) system proteins, contribute to long-term resistance in some HPV-positive HNSCC. The increased expression of *XRCC1*, *PARP1*, and DNA polymerase β enhances the repair of single-strand breaks (SSBs) and oxidative DNA damage, which are also induced by ionizing radiation. This compensatory upregulation provides a selective survival advantage, allowing tumor cells to adapt and persist despite radiation exposure. Additionally, mutations in the *XPC* gene, a key component of the nucleotide excision repair (NER) pathway, have been linked to impaired DNA repair and resistance to radiotherapy, further complicating treatment outcomes (Maćkowiak et al., 2024[53], Nickson et al., 2017[65], Weaver et al., 2015[101]).

Beyond alterations in DNA repair systems, epitranscriptomic modifications play a crucial role in regulating cellular responses to radiotherapy. *METTL3* overexpression in HPV-positive tumors stabilizes oncogenic transcripts involved in stemness, immune evasion, and DNA repair, reinforcing a pro-survival phenotype. One key target of m^6^A-mediated regulation, *SALL4* gene, plays a central role in maintaining CSC properties. By stabilizing *SALL4* mRNA, METTL3 enhances CSC survival following radiation, making these cells more resistant to DNA damage-induced apoptosis (Huang et al., 2024[35], Sun et al., 2022[88], Yu et al., 2022[112], Zheng et al., 2019[119]). 

m^6^A modification also regulates the expression of circCUX1, a circular RNA that interacts with caspase-1 mRNA, preventing its translation. Since caspase-1 is required for the activation of pro-inflammatory cytokines IL-1β and IL-18, its suppression by m^6^A-modified circCUX1 leads to reduced immune activation, allowing tumor to evade immune-mediated clearance following radiotherapy. Furthermore, m^6^A enhances the stability and translation of β-catenin mRNA, activating the Wnt signaling pathway, which has been strongly linked to therapy resistance and tumor repopulation post-radiation exposure (Wu et al., 2021[107]). 

The convergence of HPV-induced DNA repair deficiencies and m^6^A-mediated transcriptomic regulation creates a dynamic framework for adaptive radiotherapy resistance. While initial treatment response may be favorable due to defective HR repair, the upregulation of alternative repair mechanisms (BER) and the m^6^A-driven stabilization of pro-survival and DNA repair-related transcripts eventually enable tumor cells to evade radiation-induced cell death. This suggests that targeting m^6^A regulators (e.g., METTL3 inhibitors) and BER pathways (e.g., PARP inhibitors) in combination with radiotherapy may provide a more effective treatment strategy for HPV-positive HNSCC.

### Chemotherapy

Despite HPV status, HNSCC patients undergoing similar treatment regimens, accumulating evidence indicates that HPV-positive cases exhibit a better response to chemotherapy and improved prognosis compared to HPV-negative counterparts (Dolezal and Rosenberg, 2022[15]). *In vitro* studies have demonstrated increased chemosensitivity to cisplatin and enhanced radiosensitization in HPV-positive HNSCC cells. One potential mechanism underlying this phenomenon is the cisplatin-induced downregulation of viral oncoproteins E6 and E7, whose depletion promotes apoptosis, thereby increasing cellular sensitivity to treatment (Ziemann et al., 2015[123]). 

In addition, E6 and E7 have been implicated in the regulation of *SERPINB3* expression, a factor involved in the Fanconi anemia (FA) DNA repair pathway. Downregulation of *SERPINB3* in HPV-positive HNSCC leads to impaired deubiquitination of FANCD2 and FANCI, disrupting FA pathway for repairing DNA interstrand crosslinks (ICLs) induced by cisplatin (Huang et al., 2022[37]). 

Furthermore protein E7 was proven to contribute to calpain-dependent degradation. Downregulation of *AMBRA1* in HPV-positive OPSCC cell lines displays tumor-suppressive properties, positively regulates autophagy by activating ULK1 and BECN1-PIK3C3/VPS34 complexes via non-degradative ubiquitination, while also acting as a negative regulator of cell proliferation. HPV-negative OPSCC cell lines exhibit both higher AMBRA1 and autophagy levels, which contributed to their chemo-resistant phenotype (Antonioli et al., 2021[2]). 

Behind the chemotherapy resistance in cancer cells stand many well studied factors, such as: higher population of stem cells within the tumor, reduced drug accumulation, lower apoptosis levels in cancer cells and increased autophagy activity. Recently together with these factors a higher abundance in m^6^A modification is being considered to be a potential element in development of chemoresistance among HNSCC patient (Zhuang et al., 2023[121]). 

A possible example of this assumption could be a highly enriched m^6^A modification on *TRIM11* mRNA. Those m^6^A modifications were reported to increase the transcript stability, possibly contributing to an elevated *TRIM11* expression in nasopharyngeal carcinoma (NPC). Upregulated *TRIM11* was linked to the poor overall survival and higher chemoresistance of NPC cells. It is most likely caused by an overexpression of *ABCC9*, a member of the ABCC subfamily, which consists of drug transporters and multidrug resistance proteins (MRPs). Expression of *ABCC9* is regulated directly by TRIM11/Daple/β-catenin pathway, making a m^6^A modification of TRIM11 an important factor in establishing chemoresistance in tumor cells (Zhang et al., 2020[114]). 

It has been shown, that also the m^6^A methyltransferase - METTL3 may modulate chemoresistance of HNSCC cancer cells. METTL3 can be regarded as both cisplatin-sensitizing or resistance promoting factor. HNSCC *in vitro* model with *METTL3* knockdown proved to be more susceptible to cisplatin treatment showing higher percentage of cell cycle arrest in G2 phase, and decreased number of colonies formed *in vitro*, although they also presented upregulation in CSC markers such as CD44 and CD133 following cisplatin treatment (Ostrowska et al., 2024[68]). 

The m⁶A demethylase ALKBH5, regulated by the human DEAD-box RNA helicase DDX3, shows elevated expression in OSCC cell lines and may contribute to cisplatin resistance. DDX3 plays roles in promoter regulation, translation initiation, and RNA nuclear export. In chemoresistant OSCC, overexpression of DDX3 leads to increased expression of the transcription factors *FOXM1* and *NANOG*. Both genes are significantly upregulated in resistant OSCC cells, and their m⁶A methylation is controlled by ALKBH5. Consequently, the enhanced expression of *FOXM1* and *NANOG* appears to rely on DDX3-dependent upregulation of ALKBH5, underscoring the importance of m⁶A methylation dynamics in chemoresistance (Shriwas et al., 2020[84]). 

In laryngeal squamous cell carcinoma (LSCC), reduced expression of *TMA7* correlates with poor patient prognosis, while its knockdown induces increased autophagy in cancer cells. Mechanistically, the m⁶A reader IGF2BP3 binds the m⁶A-modified *TMA7* transcript and enhances its stability. This stabilized TMA7 protein directly interacts with UBA2, activating the UBA2-PI3K pathway, which in turn inhibits autophagy and promotes cell survival, migration, proliferation, and cisplatin resistance in LSCC lines (Yang et al., 2023[110]).

### Immunotherapy 

**Figure 4:**
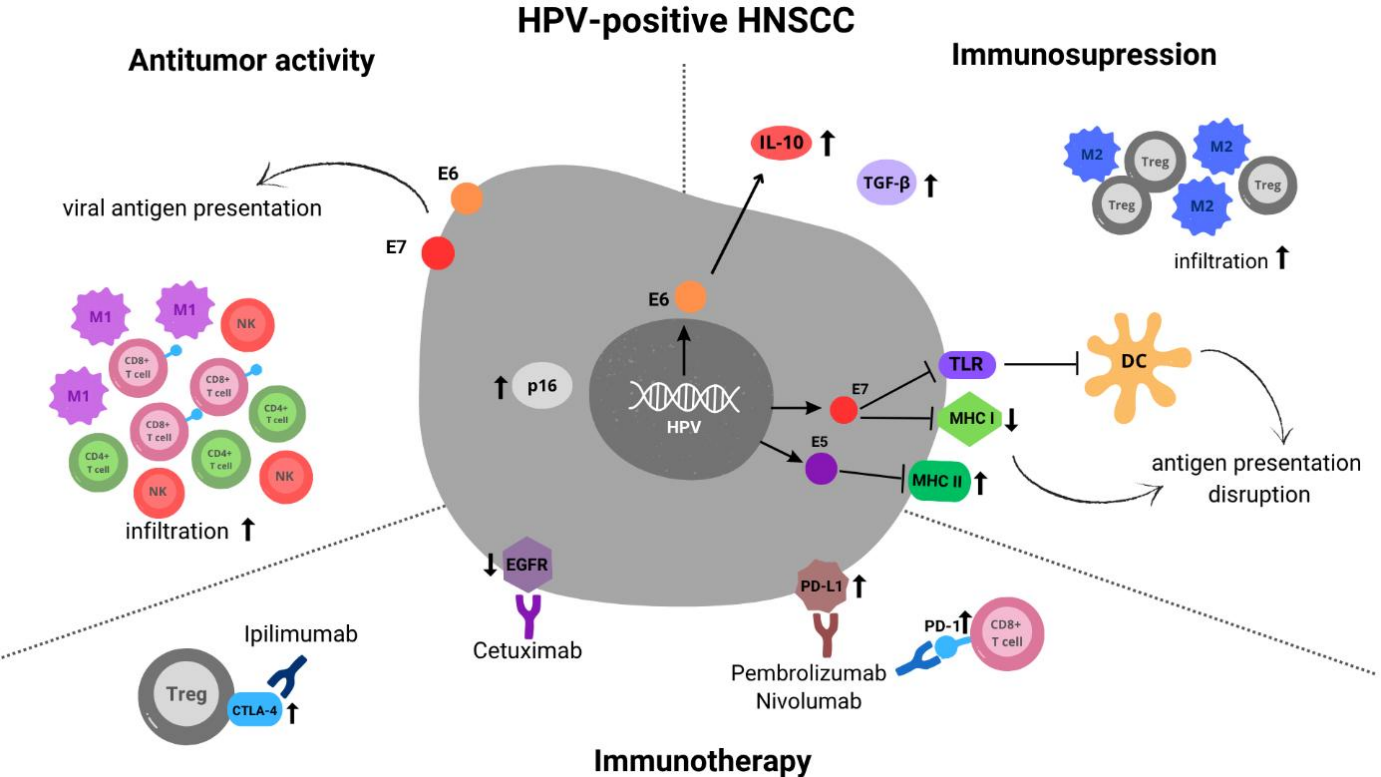
Schematic representation of components of HPV- related HNSCC microenvironment, which include: viral antigen presentation (E6, E7 HPV proteins expression), p16 overexpression, EGFR downregulation; the infiltration of anti-tumor immune cells including cytotoxic CD8+ T lymphocytes, helper CD4+ T lymphocytes, natural killer (NK) cells and M1-polarised macrophages (M1) and pro-tumor immune cells like Tregs and M2 macrophages presence; upregulation of PD-L1 and PD-1; downregulation of major histocompatibility complex class I (MHC I) expression, mediated by E5 and E7 HPV proteins with upregulation of major histocompatibility complex class II (MHC II) expression; Toll-like receptor (TLR) downregulation by E7, which further disrupts dendritic cell (DC) activation; higher CTLA-4 expression on Tregs; overexpression of transforming growth factor beta (TGF-β) and enhanced production of interleukin-10 (IL-10) mediated by E6. However HPV positive HNSCC patients are more likely to respond to immunotherapy due to upregulation of PDL, PDL1 and CTLA-4 immune checkpoints.

The immune landscape of HPV-positive HNSCC is shaped by a complex interplay between antitumor immunity and immunosuppressive mechanisms within the tumor microenvironment (Figure 4(Fig. 4)). HPV-positive HNSCC patients demonstrate significantly better overall survival and progression-free survival, with a two-fold improvement, and are 1.29-times more likely to respond to immunotherapy compared to their HPV-negative counterparts, due to the presence of viral antigens that enhance immune recognition (Wang et al., 2021[93]). 

Clinical trials such as KEYNOTE-012 and CheckMate 141 have demonstrated higher overall response rates (ORR) in HPV-positive patients treated with PD-1 inhibitors like pembrolizumab and nivolumab, both of which are currently approved by Food and Drug Administration (FDA) in HNSCC treatment. For instance, the KEYNOTE-012 study showed an 18 % ORR in HPV-positive patients compared to 8 % in HPV-negative patients. Similarly, CheckMate 141 reported a 32 % ORR in HPV-positive versus 14 % in HPV-negative patients (Wang et al., 2021[93]).

HPV-positive tumors are characterized by higher B-cells and CD8+ T-cells infiltration. Tumor infiltrating lymphocytes (TILs) (Wang et al., 2021[93]) as a part of antitumor response, are reported to be associated with survival in HPV-driven OPSCC. Chakravarthy et al. reported that CD4 and CD8 mRNA levels are significantly higher in HPV-positive OPSCC, confirming enhanced TIL infiltration (Chakravarthy et al., 2016[7]). More than 80 % of HPV-positive patients with high TIL levels exhibit a 3-year survival rate of 96 %, compared to 59 % in HPV-positive patients with low TIL levels (<20 % TILs) and 56 % in HPV-negative patients. These findings suggest that TIL density may be a predictive marker for better prognosis in HPV-positive tumors (Ward et al., 2014[100]). 

Moreover, the TME of HPV-driven tumors includes HPV-specific CD4+ and CD8+ T cells with a Th1/Th17 profile, which correlates with improved overall survival and reduced lymph node metastasis. These HPV-specific CD4+ T cells secrete cytokines such as TNFα and IFNγ, both of which promote tumor cell senescence. Notably, elevated TNFα expression is associated with enhanced therapeutic outcomes. Evidence shows that combining cisplatin with exogenous TNFα results in a higher proportion of apoptotic cells in HPV-positive cell lines, potentially contributing to improved chemotherapy responses (Welters et al., 2018[102]). 

One of the reasons for the improved response to immunotherapy in HPV-driven HNSCC is the elevated expression of immune checkpoints. CTLA-4 expression is higher in Tregs in HPV-positive tumors and may relate to increased T cell infiltration. HPV-driven HNSCC tissues also show higher PD-L1 expression, while tumor-infiltrating lymphocytes express higher levels of PD-1, both of which are associated with favorable prognosis (Wang et al., 2021[93]). 

HPV-negative HNSCC is commonly associated with traditional risk factors such as tobacco and alcohol use, resulting in a distinct tumor biology characterized by higher mutational burden and immune evasion mechanisms. Consequently, the response to immunotherapy in HPV-negative HNSCC is generally less favorable and reduced antitumor response is observed. Although the TME of HNSCC is complex, antitumor immunity is mainly driven by NK cells and effector T cells, the presence of which correlates with better patient outcomes. Conversely, poor prognosis is linked to a tumor-promoting environment shaped by regulatory T cells, neutrophils, M2 macrophages, and MDSCs. Immunosuppressive cytokines and growth factors such as IL-6, IL-10, VEGF, and TGF-β further inhibit antitumor responses (Economopoulou et al., 2016[18]).

The major mechanism of immune-escape in HNSCC is tumor antigen presentation inhibition (Wang et al., 2021[93]), due to genetic and epigenetic alterations, which decreases HLA levels and alters antigen expression (Johnson et al., 2020[42]). Also, the upregulation of PD-L1 immune checkpoint and high NK CD56+ cells infiltration, that improves a survival rates (Wang et al., 2021[93]).

Ongoing clinical trials are exploring combination therapies and novel agents to improve outcomes in HPV-negative HNSCC. The DURTRERAD trialis evaluating the feasibility and efficacy of combining durvalumab (anti-PD-L1) with tremelimumab (anti-CTLA4) and radiotherapy in locally advanced HPV-negative HNSCC. Preliminary results suggest that while the combination shows potential, toxicity remains a significant concern, underscoring the need for careful patient selection and treatment strategies (Botticelli et al., 2021[6]).

Currently, in HNSCC anti-PD-1-agents, nivolumab and pembrolizumab, are approved by FDA as a second line- treatment in recurrent/metastatic tumors. Moreover, anti-PD-1 therapy tend to be the best treatment choice for male patients, those with current and former smoking habit. In turn, anti-PD-L1 therapy might be the best treatment for never smoking patients or female patients. Considering HPV status, anti-PD-L1 and anti-PD-1 has similar probability to be effective in HPV-positive tumors, but anti-PD-1 achieve better results in HPV-negative ones (Botticelli et al., 2021[6]).

The KEYNOTE-048 study that revealed that R/M HNSCC (reccurent/metastatic) patients treated with pembrolizumab and chemotherapy had significantly higher overall survival rate compared to cetuximab and chemotherapy treatment. Based on these results, the FDA approved pembrolizumab monotherapy and pembrolizumab combined with platinum-based chemotherapy as first-line treatments for R/M HNSCC (Shibata et al., 2021[83]).

Recently, neoadjuvant immunotherapy has gained great interest in HNSCC treatment. This approach is often performed before surgical intervention, and is based on enhanced T cell systemic response for tumor-specific antigens. A phase II of randomized clinical trial (NCT02919683) showed that HNSCC patients pre-surgery treated with nivolumab (anti-PD-1 antibody) exhibit 13 % response rate whereas patients treated with nivolumab + ipilimumab (anti-CTLA-4 antibody) 38 % response rate (Huang et al., 2022[36]).

## m6A RNA Methylation and Immune Evasion

Recent studies have demonstrated that RNA methylation, particularly m^6^A, plays a significant role in modulating the immune response and the efficacy of immunotherapy. m^6^A modification in mRNA can influence the expression of immune checkpoint molecules and the presentation of neoantigens, thereby affecting tumor recognition by the immune system. Moreover, m^6^A methylation directly and indirectly regulates T cell development, homeostasis, and differentiation. For example, methylation of IL-17 mRNA in T cells promotes its translation and leads to increased IL-17 expression. In DC, the presence of m^6^A enhances DC activation and boosts their ability to stimulate T cell responses. Interestingly, a high abundance of RNA modifications such as m^6^A, m^5^C, and 2-O-methylation has been shown to suppress DC activation under necrosis-like conditions. The cross-presentation of tumor antigens and the capacity of DCs to activate T cells is enhanced when the m^6^A RNA reader YTHDF1 is deleted (Zhang et al., 2022[113]).

Upregulation of METTL3 promotes polarization of macrophages toward the M1 (antitumor) phenotype while inhibiting M2 polarization, which is associated with tumor progression (Zhang et al., 2022[113]). m^6^A-methylated RNAs also suppress activation of the innate immune system via the TLR pathway. Additionally, the expression of type I interferons, key modulators of antiviral and antitumor immunity, is regulated by m^6^A machinery: METTL14 upregulates their expression, while the demethylase ALKBH5 downregulates it (Rubio et al., 2018[79]). 

In HNSCC, the expression of the m^6^A reader YTHDC2 correlates with infiltration of CD4+ T cell subsets (Li et al., 2020[50]), and elevated PD-L1 expression has been linked with m^6^A RNA methylation levels (Yi et al., 2020[111]). These findings underscore the epigenetic role of m^6^A in shaping the immune microenvironment of tumors.

Considering the high impact of m^6^A RNA methylation in modulating immune cells activity it is fair to allow for this process as one of the key epigenetic components influencing the immune response to cancer and its treatment. Moreover, it opens up possibilities in the context of new targeted therapies and monitoring the effects of treatment.

To date, there are some studies highlight m^6^A RNA methylation role in anti-cancer therapy response. Overexpression of *YTHDF2 *gene reduces sensitivity of tumor to cisplatin in colorectal cancer. Upregulation of METTL3 in tumors is associated with tamoxifen resistance and tumor progression. In glioblastoma, temozolmide resistance is mediated in m^6^A- dependent manner. M^6^A RNA methylation related enzymes METTL3, FTO or ALKBH5 affect tumor cells metabolism, thereby causing a resistance to radiotherapy (Zhang et al., 2022[113]). In melanoma, knockdown of *FTO* resulted in increased PD-1 expression and reversed tumor resistance to anti-PD-1 treatment. What is more, combining PD-L1 checkpoint inhibition and YTHDF1 deletion, indolent tumor progression through stimulation of CD8+ T cells function (Zhang et al., 2022[113]). 

## Clinical Trials in HNSCC: Trials Focusing on HPV-Positive and HPV-Negative Tumors

Recent clinical trials based on HPV-dependent HNSCC have primarily aimed to optimize treatment by balancing efficacy with minimizing toxicity. Key studies have explored de-escalation strategies in definitive therapy, including reduced-dose radiotherapy and chemotherapy regimens, to address the unique biology and favorable prognosis of HPV-positive tumors. Many clinical trials are focusing on combining immunotherapy with traditional treatments. Novel approaches, including therapeutic HPV-targeted vaccines and agents that enhance the tumor microenvironment, are also under active investigation. These trials reflect a growing emphasis on tailoring interventions to the distinct clinical profile of HPV-related HNSCC. A summary of the latest actively conducted clinical trials is presented in Table 1(Tab. 1).

## Future Directions: Biomarkers for Personalized Treatment

Growing evidences highlights the critical role of m^6^A RNA methylation in cancer development and progression, positioning it as a potential biomarker for diagnosis, prognosis, and personalized treatment strategies. Unfortunately, there are only few data concerning epigenetic changes in RNA as a biomarker for HNSCC as a response to treatment and patient prognosis. Worth mentioning is the work by Zhao et al. focusing on a bioinformatic search for RNA methylation regulatory signatures in the prediction and prognosis of head and neck squamous cell carcinoma using TCGA data (Zhao and Cui, 2019[117]). 

In rectal cancer, m⁶A RNA methylation regulators expression have been linked to disease prognosis (Zhuang et al., 2020[122]). In lung cancer, research by Muraoka et al. suggested that m^6^A RNA methylation of miR-34b/c could serve as both a diagnostic and prognostic marker for malignant pleural mesothelioma, an aggressive lung-related malignancy (Muraoka et al., 2013[62]). 

Meanwhile, another study identified five m⁶A regulators-HNRNPA2B1, HNRNPC, KIAA1429/VIRMA, RBM15, and METTL3-as key players in determining survival rates in lung adenocarcinoma patients (Wang et al., 2021[94]). 

A study conducted in 2019 by focused on, using transcriptomic data from TCGA to analyze the correlation between m⁶A-associated risk signatures and patient outcomes. Chen et al. findings based on transcriptomic TCGA data revealed that bladder cancer patients may be classified by significant differences in survival based on the expression of *FTO*, *YTHDC1*, and *WTAP* genes (Chen et al., 2019[8]). 

More recently, Hou et al. developed a prognostic model based on m⁶A RNA methylation regulators for papillary thyroid carcinoma. This model, which incorporated *RBM15*, *KIAA1429*, and *FTO* expression, was validated as an effective predictor of overall survival in thyroid cancer patients, further cementing the role of RNA methylation in cancer prognosis (Hou et al., 2019[33]). 

With the increasing availability of RNA methylation profiling techniques, above/presented findings highlight the clinical potential of epitranscriptomic markers in precision oncology, offering new avenues for early cancer detection, risk assessment, and tailored therapeutic interventions. Furthermore, a better understanding of the role of m^6^A RNA methylation in HPV-positive HNSCC could result in the development of new biomarkers for early detection and prognosis (Minor et al., 2012[58], Misawa et al., 2020[60]). 

## RNA Methylation as a Therapeutic Target

Recent advances in cancer research highlight the potential of targeting HPV oncoproteins and m^6^A RNA methylation pathways as innovative therapeutic strategies. One of the most promising approaches in HPV-related cancers involves direct inhibiting the viral oncoproteins that sustain malignant cell growth. Strategies such as monoclonal antibodies and small-molecule inhibitors aim to disrupt E6/E7 interactions with host proteins, restoring tumor suppressor activity and reducing cancer cell proliferation (Ni et al., 2025[64]). Another promising avenue is RNA-based therapeutics, including short hairpin RNAs (shRNAs) and CRISPR-Cas9-based gene editing, that inhibit E6/E7 expression and trigger apoptosis in HPV-positive cancer cells (Folliero et al., 2023[24]). Additionally, emerging research suggests that immune checkpoint inhibitors targeting PD-L1 in HPV-driven tumors may enhance immune responses, allowing for better recognition and elimination of cancer cells (Li et al., 2024[49]). Furthermore, therapeutic HPV vaccines that elicit immune responses against E6 and E7 peptides have demonstrated significant preclinical potential (Folliero et al., 2023[24]). 

Beyond HPV oncoproteins, another promising target in cancer therapy is m^6^A RNA methylation related regulators. Inhibiting selected m⁶A writers, erasers and readers has been shown to increase sensitivity to chemotherapy and immunotherapy, suggesting that m⁶A modulation could enhance current treatment approaches (Pulliero et al., 2024[74]). Additionally, the combination of m⁶A inhibitors with DNA methylation drugs has been explored as a strategy to reprogram gene expression and reverse cancer-associated epigenetic changes (Durzynska et al., 2017[17]). 

Further studies highlighted the role of non-coding RNAs (ncRNAs) in regulating both HPV oncoproteins and m^6^A RNA methylation pathways. Certain lncRNAs interact with E6 and E7 proteins, influencing cancer cell metabolism and immune evasion, making them potential therapeutic targets (Ghiani and Chiocca, 2022[29]). Targeting these RNA molecules using mRNA-based drugs or antisense oligonucleotides could provide a novel way to correct epitranscriptomic imbalances in cancer cells.

Combining HPV oncoprotein inhibition and m^6^A RNA methylation modulation is a promising frontier in precision oncology. These targeted strategies not only disrupt key oncogenic pathways but also offer potential solutions to therapy resistance, which remains a major challenge in cancer treatment. 

## Conclusion

This review underscores the complex interplay between hypoxia, m⁶A RNA methylation, and HPV infection in shaping both the oncogenic landscape and therapeutic resistance in HNSCC. Each of these factors-acting individually or in synergy-modulates cellular metabolism, immune surveillance, and response to treatment.

Hypoxia contributes to tumor progression and immune evasion by stabilizing HIF-1α, altering m⁶A regulatory enzyme expression, and promoting resistance to radiotherapy and chemotherapy. At the same time, m⁶A RNA methylation dynamically regulates gene expression involved in DNA repair, immune checkpoint presentation, and treatment response. Dysregulation of m⁶A machinery can promote oncogenic pathways and mediate adaptive resistance to cisplatin, temozolomide, and even immune checkpoint inhibitors.

HPV-driven tumors represent a biologically distinct subset of HNSCC, characterized by viral antigen expression, altered immune microenvironments, and differential regulation of m⁶A-related enzymes. Notably, HPV-positive tumors exhibit greater immunogenicity and often demonstrate improved clinical responses to immunotherapy and radiation-outcomes that may be partially mediated by epitranscriptomic modifications.

Taken together, these insights reveal that hypoxia, m⁶A methylation, and HPV infection are not only biomarkers of tumor behavior but also active participants in therapy resistance. Targeting these intertwined mechanisms could lead to novel, more effective combination strategies-incorporating immunotherapy, epigenetic modulation, and hypoxia-directed interventions-for patients with both HPV-positive and HPV-negative HNSCC.

## Declaration

### Conflict of interest

The authors declare that they have no conflict of interest.

### Author's contribution

MM, JK, ZP, KO, EGK, WG, WS and KK contributed to methodology. KO, WS, and KK contributed to conceptualization, data curation, and formal analysis. MM and JK contributed to investigation and visualization. MM, JK and ZP contributed to writing - original draft. KO, WS and KK contributed to supervision, writing - review & editing.

### Funding

This work was supported by the project of Greater Poland Cancer Centre (grant number 9/09/2024/PRB/WCO/007).

### Usage of Artifical intelligence

The authors confirm that artificial intelligence tools were not used in the preparation or analysis of this manuscript.

## Figures and Tables

**Table 1:**
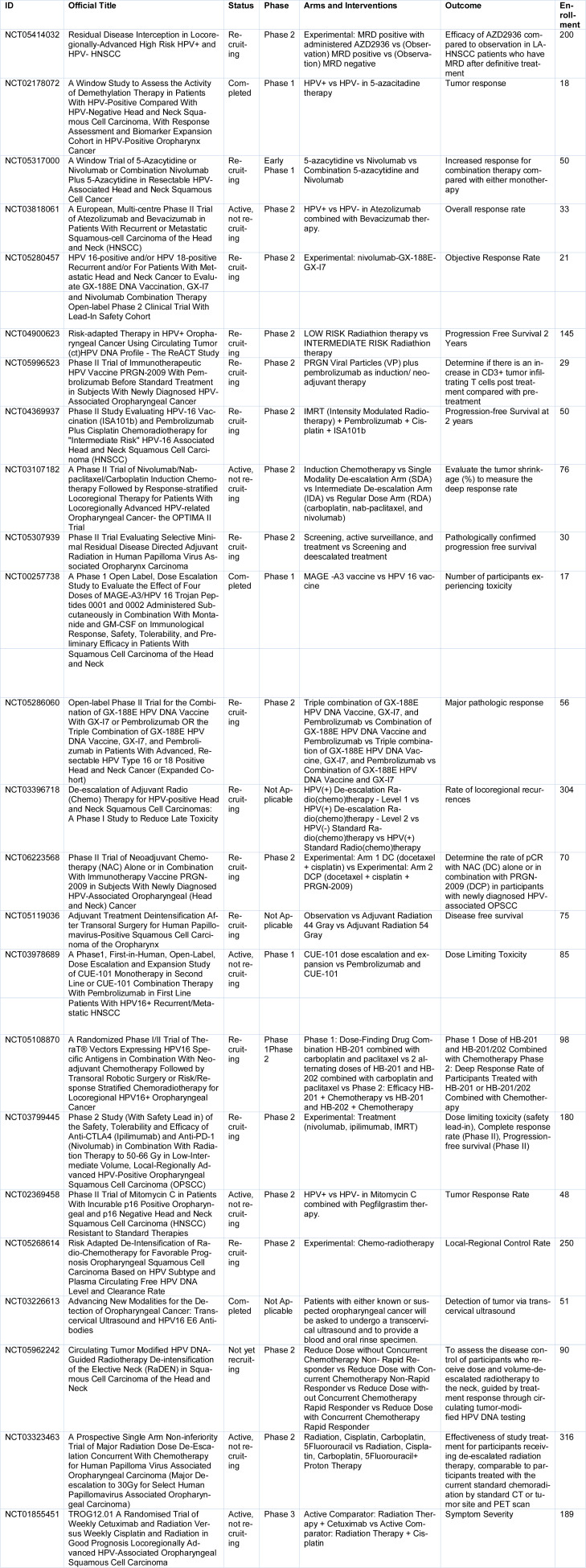
Summary of Clinical Trials Investigating HPV-Dependent Etiology in Head and Neck Cancer The table presents a selection of clinical trials evaluating different therapeutic strategies for HPV-related head and neck cancers. Including trials from various phases, ranging from early Phase 1 to Phase 2 studies. The trials explore various interventions, including immunotherapies (e.g., nivolumab, atezolizumab), combinations with targeted therapies (e.g., bevacizumab, AZD2936), and epigenetic modulators (e.g., 5-azacytidine). These trials aim to assess treatment efficacy, response rates, and overall outcomes in both HPV-positive and HPV-negative patient populations. Specific arms of the trials investigate the potential of combination therapies versus monotherapies and include both exploratory and established therapies. The details of the clinical trials are presented below.

**Figure 1:**
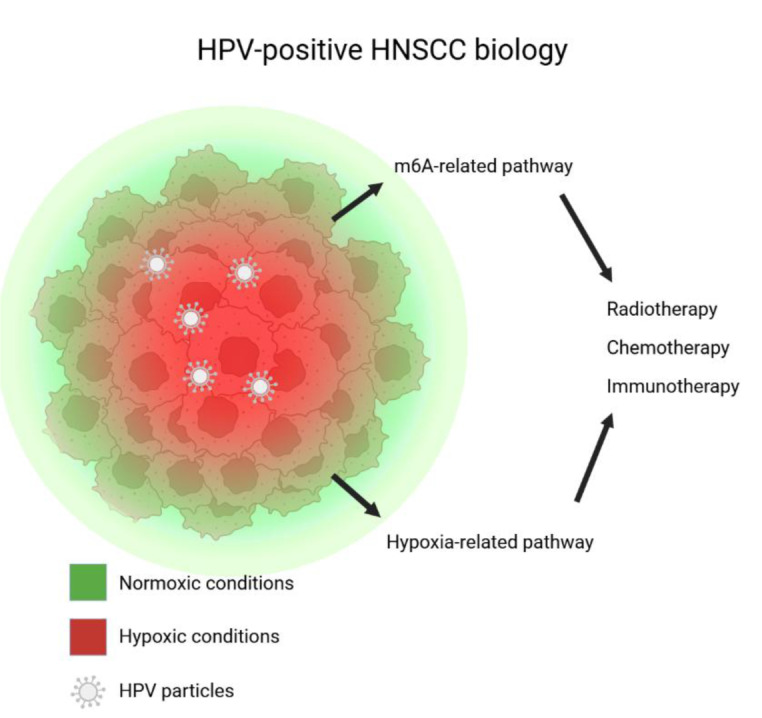
Graphical abstract. The schematic representation of a tumor microenvironment in HPV-positive HNSCC, showing the distribution of normoxic (green) and hypoxic (red) regions within the tumor mass. This review focuses on two key pathways involved in tumor progression and therapeutic response: the m^6^A related pathway and the hypoxia-related pathway. These pathways impact the efficacy of radiotherapy, chemotherapy, and immunotherapy, which are the main treatment methods for HPV-positive HNSCC.
